# Strengths and weaknesses of Problem Based Learning from the professional perspective of registered nurses^1^

**DOI:** 10.1590/0104-1169.3236.2473

**Published:** 2014

**Authors:** María Cónsul-Giribet, José Luis Medina-Moya

**Affiliations:** 2 PhD, Associate Professor, Departamento de Enfermería, Facultad de Medicina, Universidad Autónoma de Barcelona, Barcelona, Spain; 3 PhD, Full Professor, Departamento de Didáctica y Organización Educativa, Facultad de Pedagogía, Universidad de Barcelona, Barcelona, Spain

**Keywords:** Nursing, Problem-Based Learning, Curriculum, Competency-Based Education

## Abstract

**OBJECTIVE:**

to identify competency strengths and weaknesses as perceived by nursing professionals who graduated with a integrated curriculum and competency-based through Problem Based Learning in small groups.

**METHOD:**

an intrinsic case study method was used, which analyzes this innovation through former students (from the first class) with three years of professional experience. The data were collected through a questionnaire and discussion groups.

**RESULTS:**

the results show that their competency level is valued in a very satisfactory manner. This level paradoxically contrasts with the lack of theoretical knowledge they perceived at the end of their education, when they started working in clinical practice.

**CONCLUSIONS:**

the teaching strategy was key to motivate an in-depth study and arouse the desire to know. In addition, Problem Based Learning favors and reinforces the decision to learn, which is that necessary in the course of professional life.

## Introduction

The entry into the European higher education convergence process had entailed a revision of the educational paradigms towards lifelong learning. The fast changes in recent decades, in turn, have evidenced the relevance of supporting learning on the acquisition of competences that go beyond the specific knowledge in a subject area. Consequently, the teaching culture is changing from the transfer of consolidated knowledge (its admitted specialization sphere) to stimulating the students to develop analysis and synthesis skills, curiosity, critical thinking, teamwork and communicative skills, which require new teaching-learning foci.

Nursing competency is conceived as the set of skills, attitudes, knowledge and complex processes for decision making, which permit nursing activities at the required level at all times. This implies not only the ability to put the knowledge, skills and attitudes characteristic of the profession in practice to solve and prevent a health problem, but also the reference of a "know-how" that is at least structured in three dimensions: a) the conceptual dimensions or critical thinking, which implies the knowledge, decision making, analysis and formulation of problems; b) interpersonal dimensions, such as values and attitudes and c) the technical skills. All of these competences can be expressed in terms of responsibility towards the patient-user, the nurse herself, the health team, the profession, the community and the society in general.

The care practice requires that the nurses be capable of critical thinking in order to choose the best actions to solve the detected problems. In this process, learning to reason in the same way as when one is a professional is one of the most complex phases in cognitive terms and one of the themes that is most related to decision making^(^ 1 ^)^.

In this context, the Problem Based Learning proposal, PBL, has been established as a learning method whose starting point is a problem or problematized situation of real-life that permits the development of explanatory hypotheses and the identification of learning needs that allow the student to understand the problem better and achieve the previously established objectives. In addition, it permits identifying the principles related to the knowledge acquired and that can apply to other situations or problems. It is a method centered on learning, in which the students play a protagonist role^(^ 2 ^)^. PBL has evolved as a philosophy, as a form of understanding education and as a learning style^(^ 3 ^)^.

Many studies collect evidence of the PBL's contribution to teaching quality in medical studies^(^ 4 ^-^ 6 ^)^, while there exists less evidence from the nursing sphere based on the perspective of professionals who graduated in the PBL^(^ 7 ^)^.

This study departs from the innovation of a fully competency-based Nursing curriculum, which replaced classes with lectures by PBL in small groups. This experience involved the transfer from a teaching paradigm centered on the teachers and the contents of the different subjects to another one centered on the students and the basic competences and skills of the professional profile. To conclude the process, the innovation would require a general analysis of the teaching coherence in order to obtain clear criteria of improvement in the teaching and learning process.

The specific objective was focused on identifying and describing the competency strengths and weaknesses the nursing processionals who had graduated with PBL perceive.

**Figure 1:**
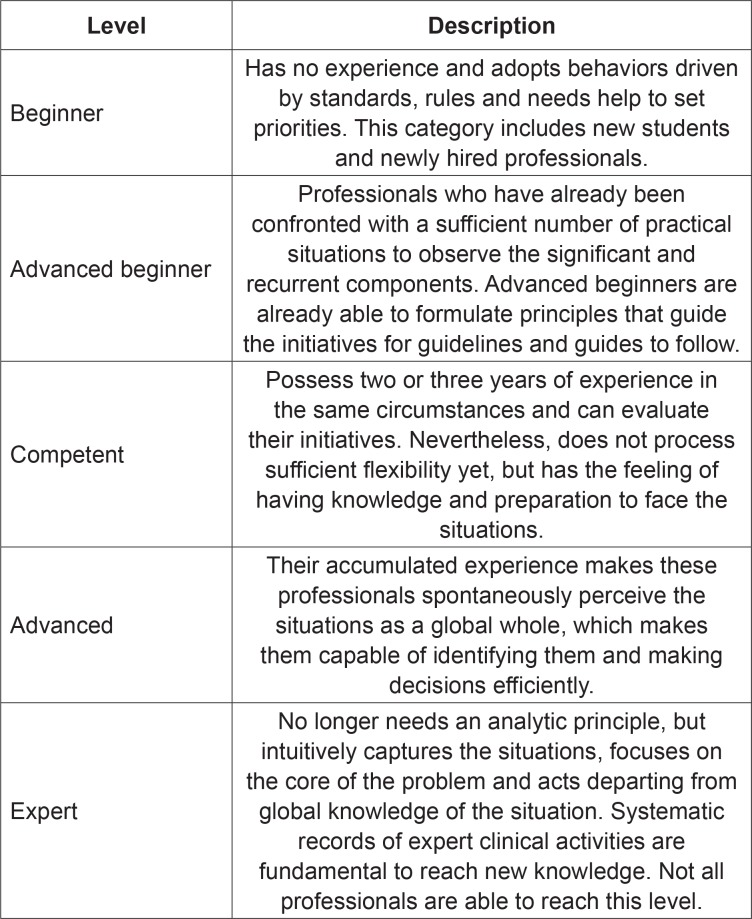
- Competency levels according to Patricia Benner

The data collection started when the professionals from the first group graduated in the PBL had obtained three years of experience; thus, they were considered in the category of "competent" nurses according to Patricia Benner's Levels of expertise^(^ 8 ^)^ (Figure 1).

## Method

An Intrinsic Case Study was chosen, as an innovative program or a school can be a case, and because both have a specific and complex function^(^ 9 ^)^. The study objective required that the participants had three years of professional experience and the graduates from the first course year that applied PBL had this characteristic. This length of experience was expected because of the reference of the "competent nurse", who according to Benner has two or three years of experience in the same or analogous circumstances and who, despite lacking the speed and flexibility of advanced nurses, has the feeling that she controls her work and is prepared to cope with and solve the different contingencies of clinical nursing.

The selection criteria were: a) the continuity in the care activity, b) the geographical proximity of their current residence and c) the availability of time.

To comply with the ethical criteria, the participants signed an informed consent form that explained the confidentiality and anonymity of their data and declarations. In addition, approval was obtained from the head of teaching and the head of care.

To collect the data, a questionnaire was elaborated and discussion groups were held. The first permitted selecting the former students, getting to know their professional reality and surveying their opinion about the influence of having graduated in a competency-based curriculum. To strengthen the open part, a numerical assessment of the competencies that guided their education process was requested, in which 1 represented deficient and 8 excellent. This allowed the participants to reflect alone, before moving on to the discussion group phase, when ideas were clarified with new questions. With the participants' permission, the discussion groups were recorded, the text was transcribed and returned to the participants for validation.

For the qualitative analysis, the constant comparison method was used, which combines the inductive category coding with the simultaneous comarison of all units of meaning found. For this purpose, the software Atlas Ti version 4.1 was used.

## Results and discussion

The researchers managed to trace and inform 81% of the graduates from the first class about the study, 33% (29 ex-students) of whom participated. Their valuation of their competences is summarized in Table 1.

**Table 1:** - Competency valuation on a scale from 1 (deficient) to 8 (excellent). Barcelona, Spain, 2008

Competency	Score	Related areas of meaning
Knowing how to work as a team	7.56	Learning among peers
		Cooperative work
Communicative skills	7.36	Interpersonal relations
Maintain updated knowledge and skills to treat new situations	7.21	Autonomous management of learning
		Motivation to learn and continue studying
		Responsibility
		Professional safety

The remaining competences run with similar mutual scores and were not accompanied by valuations that generated units of meaning: 7.12 (on 8) in the observation of ethical behavior and 6.24 (on 8) for the management of nursing care.

The weaknesses emerge from the analysis of the open question about the difficulties they have faced while working as professionals, which can be attributed to their education. The lack of theoretical knowledge clearly stands out in relation to the other difficulties that are considered characteristic of a novel nurse.

The direct influence of PBL on the way they analyze and solve their problems is acknowledged when compared to colleagues with the same professional experience and conditions. The competency strengths they describe localizes these professionals at competency level 4 (advanced), while they would actually correspond to level 3 (competent).

Concerning the teamwork, the participants' assertions confirm the opinion that the method used in their educational background strongly influences their professional activity and attitude.

...The positive part of having graduated in PBL is having worked in teams a lot, which facilitates the difficulty of working with different professionals. (...) It also helps you when you have to stand on your own feet, although asking for help is not a problem to me. I have colleagues who find it difficult to ask for help when they have any doubt. (DG*:11:7)**

This asking for help in a cooperative framework is interpreted as a value added and, in addition, is inherent in the social learning that takes place in a community of practice^(^ 10 ^)^. It is a mutual commitment for the competency of each to interact and for the achievement of objectives. The cooperation, considered as a two-way movement, is the most costly characteristic of health team work. To overcome this obstacle, it is fundamental to acknowledge that problem-based professional education, with equal responsibility for all members of the tutor group, helps to build a conception of work that is not a mere juxtaposition of subjects.

...One of the strong points of PBL is that it encourages teamwork, cooperative learning among the different members with a view to the achievement of a common goal. Teamwork is the pillar of Primary Care where I work nowadays. (IS[1]:5:65)

The quality of cooperative work in the health team requires a permanent information exchange in common spaces and times; furthermore, each professional needs to assume his/her own functions and know the other members' competences in order to avoid mixed-up roles and responsibilities.

In the last fifty years, the nursing profession has greatly advanced in strictness and method but, as a group, it maintains a culture of low self-esteem in relation to other health team professionals. The fact that the professionals from the first class that graduated with PBL feel safe in the team indicates that the thermometer of self-esteem is moving.

Interdisciplinary work demands: responsibility, dialogue skills, pact, integration, adaptation to changes, tolerance and ability to manage uncertainty. For the health professionals to consolidate the challenge of interdisciplinary dialogue as a work instrument, a symmetric position is lacking that permits the expression of each of the disciplines at an equal level^(^ 11 ^)^.

The communicative skills are part of the health professionals' core competences and are key to good care delivery to people.

...PBL facilitates the communication skills because it is not the same to study for three years listening to the teachers who tell you the concepts and a person who has sought information for three years and has contributed to the tutoring session in an argued and summarized manner. This constant communication in PBL tutoring helps a lot. (DG:11:33)

PBL tutoring in small groups is a good context to address the difficulties a student may experience with the communication skills. Difficulties that, when detected by the tutor When detected by the tutor and identified by the students, these difficulties can be addressed, supervised and improved before any contact with the patients, family members and health team in the clinical practice context.

The interpersonal relations, in turn, are the base to be able to accompany the people with health problems in their moments of weakness. These moments are unique, like each person's reactions and responses. The bonds the nurse establishes can help to establish a relation of accompaniment at times of hope and despair.

...What the PBL most contributed with for me is the improvement in my relation with the people. Before starting to study nursing, it was very difficult for me to relate and have a conversation with completely unknown people. In our profession? We always need to be in contact with people, to talk for them to understand you, to summarize concept... (IS:6:4)

In parallel, there is a generalized awareness that a generalist basic education and three years of professional experience provide neither sufficient baggage nor maturity to impregnate the interpersonal relations with humanistic and caring values - resting on the knowledge of the person, health, environment and care, always considering the emotions, emathy, compassion, authenticity, active listening, reciprocity, intuition and availability^(^ 12 ^)^. Nevertheless, it is admitted that PBL provided conditions to start.

The relations established during the tutoring sessions and the permanent peer assessment permits experiencing the relation of help established with the rest of the group members. These interactions are a source of learning of basic social skills for democratic life and for the knowledge society, in which knowing with other people also represents a core competence^(^ 13 ^)^.

By maintaining their knowledge up-to-date, the former students find themselves more autonomous to decide what care is most appropriate and it appears that the culture of technicism is making room for the wisdom of care in themes like wellbeing, adaptation and accompaniment.

...You are more autonomous when you no longer act solely as a technician, but you look for information, you move when there is a change and you do not know how to act very well (...), but you have skills to look for best evidence on how to take care and how to help. (DG:6:44)

This autonomy, which breaks with the normative strictness, can make the nursing professionals more tolerant when in biased situations, to develop creative and intellectual thinking to manage uncertainty and to apply critical thinking in view of contradictory evidence.

Autonomous learning is a competence that can be enhanced, but the conditions in which it is developed should be considered. In the participants' comments, this autonomy is linked to the motivation the fact of continuously deepening and updating one's knowledge generates.

...I am much more aware now of everything I don't know, which makes me learn continuously. PBL has granted me this "curiosity" to learn and improve. (DG:5:74)

Departing from the premise that motivation is the product of good teaching and not its requisite, it can be deduced that these professionals' motivation is intrinsic, as they profoundly focus on knowledge, with interest and a sense of importance. They observe that the strategy used has been key to that motivation and agree that teaching means stimulating the desire to have knowledge, creating teaching strategies that intensify and diversify the desire to know, something that favors and strengthens the decision to learn.

PBL furthers the sense of responsibility because it brings the students to self and mutual commitment^(^ 14 ^)^. They need to achieve personal and group objectives that will be evaluated among peers and by the tutors. Although they describe the motivation as a strength, sometimes, they refer to it as a need deriving from the lack of knowledge, as described in the paragraph about the weaknesses.

...We all know the degree of responsibility involved in going to a tutoring session. You should go prepared with a developed and internalized plan of work, so that you can contribute to your colleagues and they can contribute to you. (DG: 3:22)

The professionals graduated within this paradigm have incorporated skills and competences that will influence the future of the profession. Since, as students, they engages in the autonomous management of their learning, as professionals, they will also be more autonomous in the management of nursing care and will have elements to break with the historical role of dependence, base everything they do on evidence and gain solid knowledge based on their practice.

The graduates consider the confidence they have gained as a result of PBL as a strength but, in turn, feel more insecure to act if they are not convinced that they understand what they do. In general, there is no fear or shame in acknowledging what one does not know, on the opposite: it is considered a positive value for the safety of the persons they attend to.

...In view of symptoms or medication I do not know, or do not understand well, I have the resource I have learned through this method, which is to seek information and gain knowledge in appropriate sources. (IS:5:55)

To finish this paragraph about the strengths, the best illustration is one participant's anszer about her awareness, three years after graduating, about the paradigm change that took place in her education:

... I think that the nurse I am is the result of the way I graduated. I studied with enthusiasm, desire, curiosity, doubts, as a team and individually, with communication skills, skils to listen, ask and respect. (IS:5:75)

As regards the weaknesses, it should be mentioned that a competency-based education through PBL in small groups demands more time in comparison with large-group seminars. This means that, in the same time period, it is impossible to address the same number of themes. Due to the reduction, the student perceives gaps in the possible range of themes that is developed in traditional teaching. Nevertheless, it is considered that the addressed themes are known in depth.

...As a result of PBL, we lack knowledge in Pharmacology and Anatomy. This deficit creates great insecurity because we know much more about the diseases we studied than the people who studied with the usal method, but we do not have a clue about the diseases we did not study. (DG:11:2)

No matter the strategy used, the responsibles for teaching need to guarantee the education of competent professionals who can respond to the needs of the population. Therefore, the problems addressed in PBL should comprise all cases they will meet as professionals, and the evaluation process needs to be imbued with a strictness that guarantees that the student's final certification complies with the expected competences.

To analyze the perceived lack of knowledge, it should be kept in mind that the nurses start working immediately after graduating. At best at the services where they took their final practicums. Before gaining two or three years of experience in the same circumstances (same service), the nurses do not feel that they have sufficient knowledge and preparation to cope with the situations. Hence, it comes as no surprise that they feel a lack of knowledge until they reach the level of advanced beginner, as they have just started their professional career, without monitoring by a senior professional, and the same competency level is required from both.

The knowledge they lack is related to the basic sciences: Pharmacology, Anatomy and Physiology. Stable and descriptive knowledge the nurse should know in order to understand what happens to the patient. It is noteworthy that it is exactly Pharmacology that is perceived as the least known, given that, although the nurse should know the pharmacodynamics and pharmacokinetics to monitor interactions and adverse effects, the responsibility for drug prescription and dosage is doctors' responsibility.

...When you graduate your carreer everything is somewhat overwhelming. I arrived at the Intensive Care service and remember that, as I had neither practice nor experience, I had a lot of doubts and had a very hard time. It was logical since, independently of the way you graduated, we all depart from the reality that we are novels, we lack experience and have a lot of doubts. (DG:11:26)

In the PBL in small groups, the students will not see some themes until reaching the care practice. Nevertheless, regarding the themes they do manage, analyze and study in debt, they can assimilate the necessary principles and concepts well to face the problem and be able to extrapolate this knowledge to analogous situations. The knowledge they lack, however, add further stress to that any beginner already experiences.

## Conclusions

The safety, responsibility and skills to manage new situations, together with the highly positive valuation of their competency level, are the basic characteristics that localize the graduates at the advanced level instead of the competent level, as expected from professionals who, despite three years of professional experience, have not worked at the same service. Although this initial result is very satisfactory, it contrasts with the paradox of fitting it in with the lack of theoretical knowledge they perceived as a weakness. This contradiction makes it difficult to discern the limit between the strength of the motivation to continue studying -driven by the educational strategy PBL - and the need to gain the knowledge they say they were lacking.

The participants unanimously perceived a lack of knowledge in the basic areas Pharmacology, Anatomy and Physiology and the insecurity this perception adds to that characteristic of a novel professional, but this study does not permit distinguishing whether the perceived lack of knowledge derives from: a) superficial learning or continous learning in a spiral movement and in a profession that uses many other disciplines to oversee the results of other professionals' decisions; b) the constant scientific advances resulting from continuous recycling; c) the requirements of nursing practice, which continues putting more emphasis on the role of surveillance and cooperation with medical monitoring and treatment than on autonomous care; or d) the inability, as teachers, to coordinate and correctly drive the integration of these learnings experiences in the different problem situations.

In addition, the possibility should also be signaled that a perceived lack of knowledge in such specific areas, linked to the study of diseases and treatment, does not hide the scarce visibility of the care and the professionals who apply it in society. This invisibility strongly affect the nurses' identity and professional image. The medical treatments and technical cures have always received social and professional acknowledgement, which the accompaniment, substitution and health education the nursing professionals perform never had, and which are essential and fundamental for people's quality of life.

Finally, the evidence regarding the strength of teamwork should be evidenced in this study. The mark the acquisition of this competence leaves, which the professionals graduated with PBL acquired, should be used to the maximum in order to formulate a future proposal that approximates the team the interdisciplinary health professionals need.
